# Remarks on Mr. Charnock's Paper, and Answer to L.

**Published:** 1817-07

**Authors:** 


*For the London Medical and Physical Journal.*

*Remarks on Mr. Charnock's Paper, and Answer to L.;* by A. B., M.D.

YOUR correspondent, Mr. Charnock, in the last Number of your Journal, has not seen, probably, Dr. Murray's explanation of the active qualities of mineral waters, which are not discovered by analysis.

He should also be aware that purgatives have the good effects he mentions only in visceral obstructions of any kind, or in too high action of the system, and not in real debility of the stomach or intestines, and that if this view is not accurately preserved, many a patient will be quickly sent off the stage, some instances of which I have known. The Hamilton plan sometimes is carried too far also.

The sensations that Z. feels, who offers a premium for a solution of the causes of his symptoms, proceed from the state of the stomach, and the appearance of his urine from a too great an alkaline state of it. Should these primary remarks marks meet his eye, and he be desirous of my entering further into his complaints, without any view of a premium, he may signify his wish by addressing a few lines to you, which I shall immediately see in your Journal.

*Newcastle-on-Tyne;*

*April* 8, 1817.

